# Epidemiologic, Clinical, Ultrasonographic, and Cytological Features of Thyroid Nodules in Predicting Malignancy Risk: A Retrospective Study of 442 French Afro-Caribbean Patients

**DOI:** 10.1155/2020/4039290

**Published:** 2020-03-31

**Authors:** Johan Joseph-Auguste, Lucien Lin, Magalie Demar, Olivier Duffas, Vincent Molinie, Caroline Sulpicy, Marie-Josée Dorival, Olivier Luxembourger, Nadia Sabbah

**Affiliations:** ^1^Department of Endocrinology and Metabolic Diseases, University Hospital Center, Louis Domergues, La Trinité, Martinique; ^2^EA3593, Amazon Ecosystems and Tropical Diseases, University of French Guiana, Cayenne, French Guiana; ^3^Department of Otorhinolaryngology, University Hospital Center, Pierre Zobda Quitman, Fort-de-France, Martinique; ^4^Department of Anatomopathology, University Hospital Center, Pierre Zobda Quitman, Fort-de-France, Martinique; ^5^Anatomopathology Center, Schoelcher, Martinique; ^6^Department of General Surgery, University Hospital Center, Louis Domergues, La Trinité, Martinique; ^7^Department of Endocrinology and Metabolic Diseases, Cayenne Hospital, Andre Rosemon, French Guiana

## Abstract

**Objective:**

To evaluate epidemiologic, clinical, cytological, and ultrasonographic features of thyroid nodules in a sample French Afro-Caribbean population to determine if the standard criteria for predicting malignancy risk are applicable to this specific ethnic population. *Methods and Design*. This retrospectively designed study consisted of 442 patients who had consulted with the Endocrinology Department in Martinique (French overseas department) between 2007 and 2011. Of the 442 patients, 641 ultrasound-guided fine-needle aspirations (US-FNA) were performed by two experienced endocrinologists, and 212 patients underwent surgery. The geographical situation, age, gender of the patient, clinical and ultrasonographic features, TSH level, and US-FNA results were considered and cross-referenced with their pathology results.

**Results:**

The overall malignancy rate on final histopathology was 9% (women only), 80% of which were papillary cancer, and 20% were follicular cancer. Occult micropapillary carcinoma represented 35% of the papillary cancer. There was no significant difference in age, nodule localization, number of nodules, or thyroid function test between benign and malignant nodules. Contrary to the literature, we found only 12% incidentaloma in our series, while more than half of the nodules were discovered on palpation or as a clinical symptom. Hypoechogenicity in solid pattern nodules and nodules between 2 and 3 cm in size revealed a high diagnostic value in detecting malignancy. The corresponding rate of malignancy on Bethesda system histopathologic examination was as follows: 0% in undiagnosed (I), 0% benign (II) (micropapillary), 5% (FLUS)/atypia (III), 9% follicular neoplasm (IV), 33% suspected malignancy (V), and no malignant cytology (VI). These results show a different Bethesda system predictive value for this French Afro-Caribbean population.

**Conclusion:**

Studies evaluating ethnic cancer disparities among patients with thyroid cancer are limited and do not specifically focus on the French Afro-Caribbean population. Despite rare thyroid incidentaloma, 35% of the papillary cancer cases were micropapillary carcinoma, and the incidence and standardized mortality rate in Martinique are lower than in metropolitan France. The malignant risk distribution of thyroid FNA Bethesda classification in this sample population differs from the standard risk, and it is necessary to take that into account in the decision to operate by associating it with echographic malignancy criteria.

## 1. Introduction

Nodular thyroid disease is a common condition, especially in females and, in particular, among the elderly [1, 2]. The prevalence of thyroid cancer has increased over the last decade due to earlier detection of thyroid nodules using improved diagnostic imaging techniques. Most thyroid nodules are benign (90–95%); however, it remains difficult to detect those that may become potentially malignant. Ultrasonography is a noninvasive method that allows the practitioner to distinguish between a malignant nodule and a benign nodule. This method delivers accurate information on the nature, size, topography, echogenicity, microcalcifications, and vascularization of the nodule and therefore improves information for the fine-needle aspiration biopsy (FNAB) [3]. The ultrasound results depend on practitioner's experience. In 2007, there was no official consensus that defined the risk of cancer in relation to the ultrasonographic features of thyroid nodules. However, in 2009, in the American Thyroid Association Guidelines, fourteen sonographic features were mentioned for patients with thyroid nodules [4]. In 2011, a standardized system for analyzing and reporting thyroid ultrasound, the Thyroid Imaging and Reporting Data System (TIRADS), was used to provide initial sonographic assessment of thyroid nodules [5]. In 2017, the new European TIRADS, called EU-TIRADS, was published [6]. FNAB represents the most reliable and noninvasive exam to evaluate the risk of malignancy.

Comparative studies on thyroid cancer or nodule disparities among different ethnic groups are few, and none exist for the members of the Afro-Caribbean population living in their native environment. Several studies evaluated ethnic disparities among patients with thyroid cancer, and a few of them described clinical, epidemiological, ultrasonographic, and cytological malignancy risk in the Afro-American population [7, 8].

Environmental exposure, as well as diet and lifestyle, is known to initiate epigenetic changes. Epigenetic research in the field of thyroid cancer has contributed to improving our understanding of biologic determinants in cancer disparities [9]. Finalyson et al. described, among his study pool in England, a higher risk of thyroid cancer in black Africans and black Caribbeans compared to the white population (in particular for follicular thyroid cancer) [10].

Some studies have described a lower rate of thyroid cancer in Afro-Americans, and the data come from the US National Cancer Institute's Surveillance, Epidemiology, and End Results database from 1988 to 1992 and from 1992 to 2010 [11, 12]. Magreni et al. observed no significant difference in the incidence of thyroid cancer between whites and blacks and social determinants, and access to healthcare did not seem to be the major factor [13]. Most trials took into account racial and socioeconomic disparities [14], but few focused on epidemiologic ultrasonographic and histological differences. Moo-Young et al. showed that Afro-American patients <45 years had larger thyroid tumors but were less likely to have lymph node invasion [15]. Healthcare in Martinique is accessible to all and is therefore not a differentiation factor as it is in America. Martinique is a French Caribbean Island with 394,000 inhabitants and different ethnicities: 75% of the population is Afro-Caribbean and 25% is Caucasian. The aim of this study was to gain greater insight into potential disparities concerning the malignancy risk factors among the local Afro-Caribbean population and to describe the related epidemiologic, clinical, ultrasound, and cytological factors. The secondary objective was to analyze the cytological and histopathological correlations to determine if the Bethesda system could be applicable to this ethnic group. The results of FNAB and the ultrasonographic features were correlated with the final histopathology diagnosis after surgery. The frequency of malignancy was determined and compared with the national database.

## 2. Subjects and Methods

This study is a retrospective trial, and the study pool consisted of 530 patients referred to the Department of Endocrinology, University Hospital Center in La Trinité, Martinique. This included all patients who presented with thyroid nodules and underwent FNAB in our center between 2007 and 2009. Only two centers performing FNAB exist in Martinique. The FNAB were performed for all nodules. The criteria to refer patient in our center for ultrasound-guided FNAB were a nodule larger than 8 mm or suspicious malignant sonographic features such as microcalcification, irregular margin, markedly hypoechogenicity, and increased central vascularity.

Eighty-eight patients were excluded due to cytology or histopathology reports that were unavailable. Thyroid US and US-FNA were performed with 5–12 MHz linear array transducers (HD11, Philips-Advanced Technology Laboratories) by two specialized radiologists with over ten years of thyroid imaging experience. Ultrasonographic features were determined: nodule size, echo structure (solid, cystic, and mixed), echogenicity, margin (well-defined regular halo or poorly defined irregular), and presence or absence of microcalcifications. The vascularization was none, type I; presence of perinodular and/or slight intranodular flow, type II; and presence of perinodular and/or slight intranodular flow, type III.

Patients signed a consent form prior to the procedure. FNAB was performed after local anesthesia with xylocaine 1% gel and with antiseptic technique. The thyroid lesions were sampled from a single skin puncture with a 25G needle and, if necessary, a syringe (10 mL). Two to three samplings were performed per nodule, and the sample was air-dried. Slides prepared from each nodule were stained and fixed with alcohol and then submitted for Papanicolaou staining. When the nodule had cystic and solid components, both components were sampled. Histopathologic diagnosis was performed by four different cytopathologists during the study period. All the FNAB results were classified using the Bethesda System for Reporting Thyroid Cytopathology 2009 [16, 17]. For the FNAB performed prior to the Bethesda system, a cytopathologist reviewed and classified these samples using the Bethesda system (Bethesda classification analyzes the malignancy risk for each category: I, nondiagnostic or unsatisfactory (ND/UNS); II, benign; III, atypia of undetermined significance (AUS)/follicular lesion of undetermined significance (FLUS); IV, follicular neoplasm/suspicious for follicular neoplasm; IV, suspicious for malignancy; V, malignant). Histological examination was performed for nodules with clinical suspicion, those suspected by cytology, and patients with two consecutive AUS/follicular neoplasm diagnoses, as well as for nodules with strong suspicion of ultrasound and those measuring more than 3 cm (after agreement of the multidisciplinary consultation meeting). If a focus of microcarcinoma was found outside of the nodule of interest, it was considered clinically insignificant and was excluded for determining the predictive value of the FNAB. The following data were recorded for all the patients in the study: gender, age, residence, BMI, thyroid dysfunction, number and size of thyroid nodule, ultrasound features, thyroid stimulating hormone ultrasensitive (TSHus), and FNAB results (Bethesda system). Comparisons were performed to determine the predictive factors of malignancy. The quantitative data were obtained by calculating the mean and standard deviation. We used a student test for correlation. The qualitative data were obtained by calculating the percentage and absolute value, and comparisons were performed with a Fisher exact test for categorical variables. *p* < 0.05 was considered statistically significant.

Clinical and ultrasonographic features were evaluated using univariate and multivariate analyses with logistic regression.

### 2.1. Ethical Approval

The local ethics committee agreed, and oral consent was obtained by practitioners according to European regulations. The declaration of use of the data number 2214515 of the National Commission of Informatics and Freedoms was obtained.

## 3. Results

Between 2007 and 2009, a total of 530 patients underwent thyroid US with FNAB (exclusion criteria: nodule<8 mm); 88 patients were excluded from the analysis: 5 patients refused to be operated on, and for 83 patients, histological and operating reports were unavailable. We studied 442 patients for a total of 641 thyroid nodules. Most patients were referred by their primary care physician (66%). Of the 442 patients, 329 had an informed mode of discovery. Unlike most studies, we primarily had a clinical mode of discovery, either with palpation or during presentation of symptoms such as genes or dyspnea. In fact, this represents more than 58% of the patients in our study (20% had a thyroid history and had a follow-up). The incidentalomas represent only 12% (with those followed for parathyroid disease) of the nodules discovered, and 7% of those patients had TSH abnormalities. Among the 12 cancers, 8 were discovered on clinical signs or on palpation, 2 were incidentalomas, and 2 were nodules followed previously. Only 10% had a chance discovery after another examination, 2% were discovered during a primary hyperparathyroidism follow-up (so 12% of total incidentaloma), and 6% were discovered during a cervical ultrasound performed for biological dysthyroidism. Half of the patients (48%) were operated on to control 264 nodules and 212 patients; 22% benefited from a lobectomy and 78% from a complete thyroidectomy. Lobectomy was proposed to patients with a single nodule that was benign in cytology but had increased significantly in size (more than 20% in the year) or measuring more than 3 cm for Bethesda III (after two FNAB) or IV isolated nodules. Thyroidectomy was performed for voluminous multinodular goiter, suspicious nodule with contralateral lesion, or during lobectomy if the lesion was suspicious in extemporaneous.

The operated patients were similar to the sample patients for age, gender, TSHus rate, and personal and familial thyroid history. The body mass index (BMI) medium for operated patients with benign disease was 28, and for patients with thyroid cancer, it was 31. The patients who underwent surgery were women, who were on average 50 years old with no thyroid history. None of the patients had any familial thyroid cancer history. Most patients were female (87%), and malignant nodules were only associated with the female gender. Among the operated patients, we found a malignancy rate of 9% (*n* = 19). Microcancers were incidentally detected after thyroidectomy for another nodule in 3%. These occult micropapillary carcinomas represented 35% (*n* = 7) of the cancers. Among the cancers, 80% were papillary carcinomas, and 20% were follicular carcinomas (one microfollicular).

Patients with thyroid cancer were not significantly different from those with benign nodules (*p* > 0.05). The thyroid cancer rate increased with age until an average age of 50 years in each histological type (Table 1). Overall, 25% (*n* = 5) had a benign thyroid history, 10% (*n* = 2) had a malignant transformation (these 2 cases were those of two patients who were considered to have repeatedly benign nodules by FNAB, who were followed during 5 years for one and 4 years for the second, and in whom FNAB finally found lesions of the follicular neoplasm type justifying surgery and the ultimate diagnosis of thyroid cancer (papillary carcinoma)), 15% (*n* = 3) had a benign nodule history, and 50% had no thyroid disease history.

### 3.1. Ultrasound Description

The majority of operated goiters were 64% (*n* = 163), including nodules superior at 3 cm, 71% (*n* = 150) were plurinodular, and 29% (*n* = 62) were isolated nodules. Of these, 65% (100/153 indicated values) had a solid echo pattern, 31% (48/153 indicated values) had a mixed echo pattern, and 11% (22/194 indicated values) had hypervascularization type III. Of the patients, 63% (141/223 indicated values) were hypoechogenic, 20% (53/262 indicated values) had irregular borders, and 20% (54/264 indicated values) had microcalcifications (Table 2). At the time of the study, the TIRADS classification was not still in use, and in the data alone, the echogenicity, irregularity of the margins, vascularization, and the presence of microcalcification were described. There was little information in the records about height and width, as well as the importance of hypoechogenicity. Thus, we could not reclassify the nodules in TIRADS but only describe the echogenicity, the limits, the presence of calcifications, and the type of vascularization. Tumors with sizes <3 cm and >2 cm were significantly more prevalent for a malignant nodule (*p* < 0.05). Plurinodular goiters were not associated with a cancer risk. The ultrasound criteria of malignancy that stood out from the univariate logistic regression were the irregular borders (*p* < 0.05) (Table 2).

## 4. FNAB

Our cytology results found 45% of tumors were benign cytology (Bethesda II), 16% were atypia of undetermined significance (Bethesda III), 27% were follicular neoplasms (Bethesda IV), and 5% were suspected of malignancy (Bethesda V) with respective risk of malignancy of 0%, 5%, 9%, and 33%, respectively (Table 3). We did not find malignant cytology (Bethesda VI). Among the operated patients who underwent FNAB, 14% of the cytologies were not contributory, and mostly solid nodules were found. Of these, none were malignant, and 81% of these cytologies corresponded to nodules larger than 3 cm. Cytological results did not agree with histological results in 11% of cases. Of these cytological discordances, 60% were nodules larger than 3 cm. The reliability of cytology decreased with nodule size (Table 4).

In thyroid cancer nodules (excluded occult micropapillary) with suspicious malignancy cytology (Bethesda V), hypoechogenicity was found in 75% of tumors, irregular margin was found in 25%, microcalcifications were found in 16%, and vascularization III was found in 33%. The concordance between Bethesda classification and ultrasound criteria in thyroid cancer is described in Table 5. The cytology in our study had a sensitivity of 83% and a specificity of 72% (noncontributive cytology was included, and occult micropapillary carcinomas were excluded from the results).

## 5. Discussion

The incidence of thyroid cancer has increased since the 1990s. It is now the 5th most common cancer in women, whereas 20 years ago, it was 14th [18, 19]. The incidence of thyroid cancer in Martinique is 2.2 for men and 8.6 for women per 100,000 inhabitants; in metropolitan France, the figures are 4.89 for men and 14.8 for women, per 100,000 inhabitants. The standardized mortality rate is also lower, at 0.2 and 0.25 for Martinique and metropolitan France, respectively (data from the general cancer registry of Martinique). Our results confirm previous studies showing a different incidence and survival among people of different racial and ethnic backgrounds, such as study by Morris et al., who described a lower incidence of thyroid cancer for the Afro-American population [20]. Tang et al. in 2018 showed no differences for disease-specific survival but different risk factors between black and white patients. In this study black Americans had less lymph node metastasis of classical variant papillary thyroid carcinoma and follicular variant papillary thyroid carcinoma but not follicular thyroid carcinoma and had a higher risk of distant metastasis for a follicular variant papillary thyroid carcinoma [21]. A recent study described a higher thyroid cancer incidence in the black population, which was due principally to an increased risk of follicular cancer. In contrast, among the Chinese population, the main risk was papillary cancer [10]. Currently, the etiologies of the global thyroid cancer increase remain unknown, but environmental and hormonal influences are possible factors, even if the increase in detection is partly due to the rise of incidentaloma screening, thanks to advances in ultrasound and CT imaging. In our study, most patients were females (87%), and malignant nodules were only associated with females. In the literature, some studies point to a potential risk of thyroid cancer in obese patients [22, 23]. In our study, the average BMI in operated men was 25, while women's BMI was 28, and for those with benign histology and those with cancer, it was 31. Unfortunately, data about weight and height were incomplete, so only 130 of 220 surgical patient charts were informed. In 2019, Kwon et al. described a higher risk of thyroid cancer in obese men and excessive adiposity per se as an independent risk factor, while women have a greater risk only when obesity is associated with metabolic abnormalities [24]. The PODIUM study showed a very high prevalence of women's obesity: 27% versus 16% for men in Martinique, which could be one hypothesis for this high female prevalence [25]. In our region, unlike other French departments, the discovery of nodules is mainly clinical, and they are detected in the course of treating a different complaint, with cervical palpation or complaint (cough, pharyngeal discomfort, voice modification, or lymphadenopathy) near 50%. Brito et al. showed that, between 1935 and 2012, thyroid cancer in Olmsted County, Minnesota, had an increased incidence of thyroid cancer to 7.1 per 100,000 persons per year between 1990 and 1999 and 13.7 per 100,000 persons per year during 2000–2012, principally due to the increase in occult cancers accidentally discovered (20% of cases), but disease mortality was unchanged [26]. The radiological prevalence of incidentaloma in Martinique is low (10% incidentaloma in our study versus 55% in metropolitan France) [27]. This explains the late discovery of nodules and the fact that more than half of them measured over 3 cm (61%). This delay could be related to the difficult access to radiological examinations in Martinique, due to the limited number of radiologists (9 per 100000 inhabitants in Martinique and 11 per 100000 inhabitants for France hexagonal) and a smaller number of endocrinologists (2 per 100000 inhabitants compared to France hexagonal 2.6 per 100000) http://www.data.drees.sante.gouv.fr. This is contrary to what is described by Uldelsman and Zhang, who linked the increase in the incidence of thyroid cancer to the increase in endocrinologists and surgeons in the United States who perform ultrasonography and subsequently FNAB, more often [28]. As described by Reitzel et al. [29], it is important to limit the indications of thyroid cancer, especially in populations where access to care is easy and where the overdiagnosis of thyroid incidentaloma is responsible for the increase in thyroid cancer. In 2006, an American team published a study on the risk of malignancy in different populations according to the social criteria. It found a bias in the analysis of the prevalence of thyroid cancer in populations based on ease of access to the healthcare system, such as the socially disadvantaged [30, 31]. In addition, the patient's noncompliance with different screening campaigns could also be involved. In our study, access to care is the same for the majority of the population because all patients have access to social insurance. However, there may be a bias due to the access to specialists, which is more complicated for residents of municipalities located far from major cities and who have little public transport. Despite this delay in access to healthcare, we observed that, in our population, papillary cancer is more frequent. We suppose that the higher iodine levels on our island could contribute to this result (no data exist for French Caribbean islands on median urinary iodine concentrations). Insufficient iodine in the diet is associated with an increase in the risk of follicular thyroid cancer; in contrast, a diet high in iodine, such as in the Caribbean islands, has been associated with an increased risk in papillary thyroid cancer [32]. We observed 35% of incidental micropapillary cancers (as defined by the WHO, any thyroid papillary carcinoma of less than 1 cm in diameter) [33]. Currently, there is no clear definition for incidental thyroid carcinoma, and thus, we observed a very different percentage than the literature [34]. Most patients have a very good prognosis, and the outcome for these patients is excellent. Many micropapillary cancers were discovered during the autopsy of patients who died from an etiology other than thyroid cancer [35, 36].

In our study, we obtained a thyroid FNAB in the group of patients who underwent an operation and classified them using the Bethesda system nondiagnostic (I) 36 (14%), benign (II) 111 (45%), atypia of undetermined significance/follicular lesion of undetermined significance (III) 41 (16%), follicular neoplasm/suspicious for a follicular neoplasm (IV) 57 (27%), suspicious for malignancy (V) 15 (5%), and no malignancy (Table 3). We observed a different predictive malignant risk for thyroid FNAB in the Afro-Caribbean population using the Bethesda system (Table 3). A meta-analysis by Sheffield et al. demonstrated a wide variety of observed nodule malignancy risks, particularly for Bethesda III and Bethesda IV [37]. Archuletta et al. found no significant differences in the risk evaluation for individual Bethesda categories between Afro-Americans and non-Afro-Americans but observed a higher number of cases in Bethesda III. They described more follicular carcinoma and less papillary carcinoma than in the white population and larger tumors at initial diagnosis [7], but Bresler et al. found that the nodule's malignancy risk for Bethesda III and Bethesda IV was nearly twice the expected risk based on original Bethesda system reports [8]. Royer et al. described a source of artifacts from ultrasound gel medium, which could lead to misdiagnosis, but in our study, we used probe cover to limit this risk [38]. We have noticed two nodules with malignant transformation. Obviously, this could have been a repeated false diagnosis of benign lesion, while it was in fact already a malignant. However, such malignant transformation of a benign nodule has already been described in the literature particularly because follicular adenomas and Hürthle cell adenomas have similarities with both benign and malignant tumors, suggesting that some of these lesions are premalignant. They usually have a slow and favorable evolution [39]. A new entity described in 2016, the noninvasive follicular thyroid neoplasm with papillary-like nuclear features, which is a subset of follicular variant of papillary thyroid carcinoma with an indolent evolution [40] could also have been evoked. Unfortunately, in our study, we could not do a screening to search for noninvasive follicular thyroid neoplasm with papillary-like nuclear features, because the period of time between the publication and the pathology was too long, which might have changed the malignancy rate in our study [41]. Concerning the absence of Bethesda VI cytology specimens, it could be due to the fact that the diagnoses were performed by general pathologists and not by specialized cytopoathologist.

Concerning the weak points of this study, our data are not fully representative of the total population, and the results could be biased due to the low proportion of patients from the North Caribbean. In addition, information on education, drinking and smoking habits, occupation, and occupational and recreational exposure to chemicals is incomplete, and information about the nodule shape (taller than wide) for nodule malignancy risk evaluation was not available, so TIRADS classification was not applicable. The small number of cancers limits the statistical analysis; in fact, we mainly note the hypoechogenicity as an echographic criterion almost always present (75% of thyroid cancer), whereas the irregular limits or microcalcifications are present, respectively, only in 25 and 16% (Table 5). Another limit to our study is the time elapsed from 2009 to the present; however, the population in our island has remained stable and the environmental factors and the situation of the number of radiologists and specialists have not changed.

## 6. Conclusion

Clinical, cytological, and ultrasonography characteristics of thyroid nodules to evaluate the risk of thyroid cancer malignancy in different racial groups cannot be clearly identified. In this French Afro-Caribbean population, some elements have been highlighted: the incidence among women is greater than in other departments, micropapillary and papillary carcinoma predominate, and the risk assessment of malignancy by the Bethesda system is less reliable for groups IV and V. The prevalence of thyroid cancer in Martinique is lower, with a mortality rate identical to that of other French departments. Thus, it is intriguing to note that symptoms and clinical examinations remain the preferred mode of screening and avoid having to question the management of incidentalomas.

## Figures and Tables

**Table 1:** Characteristics of the patients who underwent surgery.

Variables	Patients without surgery (*n*^*∗*^ = 442)	Patient with surgery (*n*^*∗*^ = 212)	*p*
Age (years)	51 ± 14	50 ± 13	0.6
Sex			0.3
Women	398 (90%)	185 (87%)	
Men	44 (10%)	27 (13%)	
Family history			0.8
None	414 (94%)	198 (93%)	
Nodule	28 (6%)	14 (7%)	
Thyroid cancer	0 (0%)	0 (0%)	
Personal thyroid history			0.7
None	354 (80%)	168 (80%)	
Cytology	44 (9%)	15 (7%)	
Previous surgery	34 (8%)	19 (9%)	
Thyroid disease	14 (3%)	7 (3%)	
TSH	1.3 ± 2	1.2 ± 2.3	0.6
Geographical distribution			0.8
Center	139 (31%)	65 (31%)	
South	95 (21%)	38 (18%)	
North-Atlantique	189 (43%)	94 (45%)	
North-Caribbean	12 (3%)	6 (3%)	

^*∗*^Number of nodules.

**Table 2:** Ultrasound characteristics: echogenicity, irregular margin, and microcalcification in 414 thyroid cancer and benign lesions.

		Histology benign (*n* = 261)	Histology malignant (*n* = 12)	*n*	*p*
Hypoechogenicity, *n*	Iso	73 (35%)	2 (17%)	75	0.22
	Hyper	6 (2.8%)	1 (8.3%)	7	—
	Hypo	132 (63%)	9 (75%)	141	—

Irregular margin, *n*	No	201 (80%)	6 (55%)	207	
	Yes	48 (20%)	5 (45%)	53	0.05

Microcalcifications, *n*	Yes	51 (20%)	3 (25%)	54	0.71
	No	203 (80%)	9 (75%)	212	—

**Table 3:** Malignancy risk and Bethesda classification.

Cytological diagnosis Bethesda classification	All FNAB, *N* (%)	All FNAB with surgical follow-up, *N* (%)^*∗*^	Malignancy risk, *N* (%)^*∗∗*^	Benign histology,^*∗∗*^*N* (%)
Benign II	347 (55%)	111 (45%)	0 (0%)	111 (100%)
AUS/FLUS III	60 (9.5%)	41 (16%)	2 (5%)	39 (94%)
Follicular neoplasm IV	79 (12.5%)	57 (27%)	5 (9%)	52 (91%)
Suspicious of malignancy V	15 (2.5%)	15 (5%)	5 (33%)	10 (66%)
Nondiagnostic I	115 (18.5%)	36 (14%)	0 (0%)	36 (100%)
Total	616	260	12	248

^*∗*^Number and percentage of cases operated in each diagnostic category; ^*∗∗*^number and percentage of cases calculated from the total number of operated cases in each category. AUS/FLUS: atypia of undetermined significance or follicular lesion of undetermined significance.

**Table 4:** Concordance between noncontributory FNAB and nodule size.

FNAB	Concurring	No concurring	Non contributory
Nodule size (cm)			
<2	17 (6%)	6 (2%)	2 (1%)
2-3	60 (22%)	6 (2%)	5 (2%)
>3	121 (44%)	18 (7%)	29 (11%) *p* < 0.03

**Table 5:** Concordance between Bethesda and echogenicity in the thyroid cancer group.

Bethesda classification	Hypoechogenicity^*∗*^	Irregular margin^*∗∗*^	Calcifications^*∗∗∗*^	Vascularization type III^*∗∗∗∗*^	Total cancer
Benign II	0	0	0	0	0
ASI/LFI III	2 (100%)	0	1 (50%)	1 (50%)	2
Follicular neoplasm IV	3 (60%)	1 (20%)	0	0	5
Suspicious of malignancy V	4 (80%)	2 (40%)	1 (20%)	3 (60%)	5
Nondiagnosed I	0	0	0	0	0
Total	9 (75%)	3 (25%)	2 (16%)	4 (33%)	12 (100%)

^*∗*^Number of nodules in each category of echogenicity; ^*∗∗*^number of nodules in each category with irregular margins; ^*∗∗∗*^number of nodules in each category with calcifications; ^*∗∗∗∗*^number of nodules in each category with vascularization type III. AUS/FLUS: atypia of undetermined significance or follicular lesions of undetermined significance.

## Data Availability

The data that support the findings of this study are available from the corresponding author upon reasonable request.

## Abbreviations

TIRADS: Thyroid Imaging and Reporting Data System
FNAB: Fine-needle aspiration biopsy
ND/UNS: Nondiagnostic or unsatisfactory
AUS: Atypia of undetermined significance
FLUS: Follicular lesion of undetermined significance
TSHus: Thyroid stimulating hormone ultrasensitive
BMI: Body mass index.
